# Enhancing Human Cutaneous Wound Healing through Targeted Suppression of Large Conductance Ca^2+^-Activated K^+^ Channels

**DOI:** 10.3390/ijms25020803

**Published:** 2024-01-09

**Authors:** Chang-Rok Choi, Eun-Jin Kim, Tae Hyun Choi, Jaehee Han, Dawon Kang

**Affiliations:** 1 Department of Physiology, College of Medicine, Gyeongsang National University, Jinju 52727, Republic of Korea; choicr63@gmail.com (C.-R.C.); eunjin1981@gnu.ac.kr (E.-J.K.); jheehan@gnu.ac.kr (J.H.); 2 Institute of Medical Sciences, Gyeongsang National University, Jinju 52727, Republic of Korea; 3 Thenevus Plastic Surgery Clinic, Seoul 07013, Republic of Korea; psthchoi@hanmail.net

**Keywords:** cutaneous wound healing, human neonatal dermal fibroblast, large conductance Ca^2+^-activated K^+^ channel, normal human epidermal keratinocyte

## Abstract

The modulation of K^+^ channels plays a crucial role in cell migration and proliferation, but the effect of K^+^ channels on human cutaneous wound healing (CWH) remains underexplored. This study aimed to determine the necessity of modulating K^+^ channel activity and expression for human CWH. The use of 25 mM KCl as a K^+^ channel blocker markedly improved wound healing in vitro (in keratinocytes and fibroblasts) and in vivo (in rat and porcine models). K^+^ channel blockers, such as quinine and tetraethylammonium, aided in vitro wound healing, while Ba^2+^ was the exception and did not show similar effects. Single-channel recordings revealed that the Ba^2+^-insensitive large conductance Ca^2+^-activated K^+^ (BK_Ca_) channel was predominantly present in human keratinocytes. NS1619, an opener of the BK_Ca_ channel, hindered wound healing processes like proliferation, migration, and filopodia formation. Conversely, charybdotoxin and iberiotoxin, which are BK_Ca_ channel blockers, dramatically enhanced these processes. The downregulation of BK_Ca_ also improved CWH, whereas its overexpression impeded these healing processes. These findings underscore the facilitative effect of BK_Ca_ channel suppression on CWH, proposing BK_Ca_ channels as potential molecular targets for enhancing human cutaneous wound healing.

## 1. Introduction

Cutaneous wound healing (CWH) is a complex, multi-step process involving several cell types, including epidermal keratinocytes, fibroblasts, endothelial cells, and peripheral nerve cells. A critical phase in this process is re-epithelialization, which entails the migration and proliferation of keratinocytes from the surrounding epidermis [1]. This phase is characterized by keratinocytes at the wound margin proliferating behind actively migrating cells, forming a dense hyperproliferative epithelium visible as migrating cell sheets at the wound margins [1,2]. Fibroblasts collaborate with keratinocytes at the wound site, and this interaction is primarily regulated by growth factor receptors, integrins, the extracellular matrix, and extracellular and cell surface proteases [3,4].

Numerous studies have emphasized the role of K^+^ channels in wound healing. These channels play a pivotal role in cell proliferation, apoptosis, and differentiation [5,6,7,8]. K^+^ channel blockers can inhibit cell proliferation and migration in a variety of mammalian cells, a process initiated by wound healing [9,10]. Conversely, K^+^ channel inhibition has been found to accelerate wound healing in intestinal epithelial cells [9,11]. Keratinocytes express various K^+^ channels, including ATP-sensitive, two-pore domain, and Ca^2+^-independent/dependent K^+^ channels [12,13,14,15]. However, the modulation and function of these channels in normal human epidermal keratinocytes (NHEKs) remain underexplored.

Our study hypothesized that modulating K^+^ channels in human keratinocytes, which are major cell populations in the epidermis, could affect cell proliferation and migration during CWH. We investigated the expression of K^+^ channels in NHEKs and examined how their modulation affects CWH.

## 2. Results

### 2.1. Facilitated In Vitro and In Vivo Wound Healing by High Concentrations of KCl

**Figure 1:**
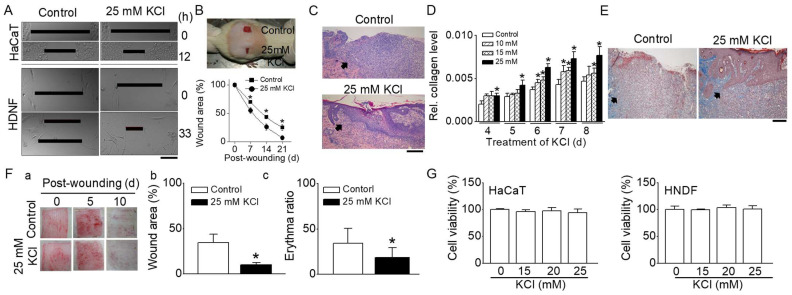
Effect of high concentrations of KCl on cutaneous wound healing. (**A**) In vitro wound healing assays in HaCaT cells and HNDFs. Enhanced wound closure in cell monolayers of HaCaT and HNDFs treated with 25 mM KCl at 12 and 33 h, respectively (*n* = 6). (**B**) In vivo wound healing assays in rats. Upper panel: Two full-thickness wounds (2 cm diameter) were created on the backs of rats. Wounds were treated with either 5 mM KCl (control) or 25 mM KCl. A representative photograph on day 14 post-wounding is shown. Lower panel: Quantitative analysis of wound areas; plotted as a percentage of the initial area measured at day 0 (*n* = 6). The graph demonstrates a significant reduction in wound size in the 25 mM KCl-treated group compared to controls. (**C**) Histologic examination (H&E Staining) of cutaneous wounds in rats. The histological sections show the epithelialization of the epidermis (indicated by arrows) in rat skin wounds (*n* = 3). (**D**,**E**) Effect of high concentrations of KCl on collagen levels in human neonatal dermal fibroblasts (HNDFs) and collagenesis in rat skin (*n* = 3). Analysis of collagen levels in HNDFs treated with high concentrations of KCl (**D**). Masson’s trichrome staining of rat skin, indicating collagen (marked by arrows) (**E**). (**F**) In vivo wound healing assays in pigs (*n* = 3). (**F**(**a**)) Representative photographs of wounds at days 0, 5, and 10 post-wounding. (**F**(**b**,**c**)) Comparative analysis of wound area and erythema ratio between control and 25 mM KCl-treated wounds. (**G**) Cell viability of HaCaT cells and HNDFs in response to high concentrations of KCl (*n* = 3): cells were treated with various concentrations of KCl for 24 h. The data are presented as mean ± SD from three to six independent experiments. Asterisks indicate a significant difference from control values (*p* < 0.05). All scale bars represent 200 μm.

To identify the influence of K^+^ channels on the healing of wounds, a concentration of 25 mM potassium chloride (KCl) was applied to wounds created mechanically. This increase in extracellular K^+^ to 25 mM leads to depolarization of the plasma membrane and inhibits K^+^ channels, which is similar to the effect of a K^+^ channel blocker. The healing process was examined in vitro using monolayers of HaCaT (an immortalized human epidermal keratinocyte cell line) and human neonatal dermal fibroblasts (HNDF), as shown in Figure 1A. A defect approximately 600 µm wide was created by scraping the cell layers. The closure of wounds in HaCaT cells improved by about 24% at 12 h and in HNDF cells by approximately 56% at 33 h with 25 mM KCl, in comparison to the corresponding control groups.

An in vivo study using a rat model also demonstrated that wound healing was expedited with 25 mM KCl compared to the control group (5 mM KCl), as shown in Figure 1B. In the group treated with 25 mM KCl, the wound sizes at 7, 14, and 21 days post-injury were significantly smaller than those in the control group (wound area: 70 ± 1, 43 ± 3, and 25 ± 5% in the control versus 55 ± 5, 26 ± 6, and 7 ± 3% in 25 mM KCl). The group treated with 25 mM KCl exhibited more complete and mature epithelialization of the epidermis, as observed through H&E staining (Figure 1C).

A collagen assay indicated that higher KCl concentrations increased collagen levels over time in HNDFs post-wounding (Figure 1D). Particularly, the collagen levels during wound healing were significantly higher in the 25 mM KCl treatment than in the controls, even in the short term (*p* < 0.05). Masson’s trichrome staining revealed denser and more horizontally aligned collagen in the 25 mM KCl-treated group compared to the control (Figure 1E). A wound healing assay was also conducted on pigs, given the anatomical and physiological similarities of pig skin to human skin. The in vivo pig model showed enhanced wound healing in the 25 mM KCl-treated group at 5 days post-wounding compared to the control group (wound area: 43.4 ± 9.2% in control versus 20.3 ± 2.4% in 25 mM KCl, Figure 1F(a,b)). Erytema is a common skin response characterized by redness, which can be due to increased blood flow in superficial capillaries. This response often occurs as part of the inflammatory process, which is a natural part of wound healing. The average erythema ratios at 10 days post-wounding were 34.2 ± 16.3% in the control and 18.5 ± 10.8% in the 25 mM KCl-treated groups, respectively (Figure 1F(a,c)). The scars in the 25 mM KCl-treated group were significantly more mature (less red) than those in the control group (*p* < 0.05). Figure 1G presents the cell viability of HaCaT cells and HNDFs in response to high KCl concentrations. The viability percentages for HaCaT cells treated with 10, 15, and 25 mM KCl were 95.9 ± 3.8, 97.6 ± 5.8, and 94.0 ± 6.8%, respectively. For HNDFs treated with 10, 15, and 25 mM KCl, the viabilities were 99.5 ± 1.1, 103.6 ± 4.8, and 101.2 ± 6.2%, respectively. In both HaCaT cells and HNDFs treated with KCl, no significant differences were observed compared to controls, suggesting that KCl concentrations ranging from 10 to 25 mM are not cytotoxic to these cells (*p* > 0.05).

### 2.2. Effect of K^+^ Channel Blockers on In Vitro Wound Healing

**Figure 2:**
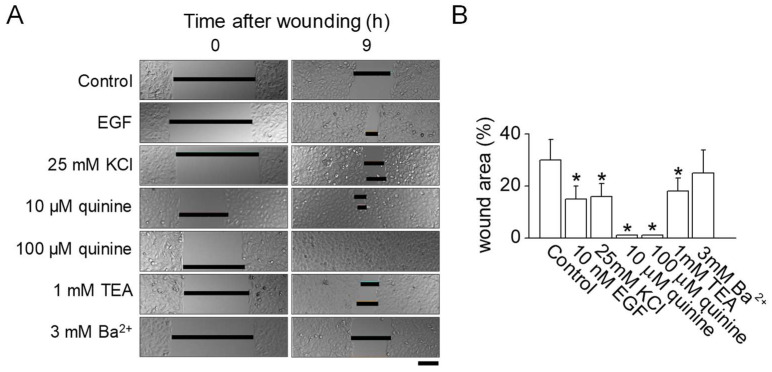
Effect of inhibiting K^+^ channel activity on in vitro wound healing. (**A**) Normal human epidermal keratinocytes (NHEKs) monolayer wound healing assay. NHEKs were grown to form monolayers, which were then mechanically wounded by scraping. Following this, the cells were immediately exposed to fresh growth media, either with or without the addition of various K^+^ channel blockers. These blockers were used at concentrations determined to be non-cytotoxic to the cells. The wound area was quantitatively measured 9 h after the wounding procedure. Scale bar, 50 μm. (**B**) A summarized data analysis of the effect of K^+^ channel blockers on the wound healing process in NHEKs. The data are presented as mean ± SD from five independent experiments. * *p* < 0.05 compared to control.

The application of 25 mM KCl for inhibiting K^+^ channels was found to promote wound healing both in vitro and in vivo (Figure 1). To verify the connection between K^+^ channel inhibition and wound healing in normal human epidermal keratinocytes (NHEKs), various K^+^ channel blockers were evaluated. As shown in Figure 2, wounds in NHEKs treated with either 10 or 100 µM quinine exhibited significantly better healing compared to untreated control NHEKs (*p* < 0.05). In a manner akin to the use of epidermal growth factor (EGF) as a positive control, wound healing was enhanced by 14% with the application of 25 mM KCl compared to controls. The use of 1 mM tetraethylammonium (TEA) also resulted in notable wound closure when compared to the control group (*p* < 0.05). However, 3 mM Ba^2+^, commonly used as a blocker for voltage-dependent K^+^ channels, did not show any improvement in wound healing (Figure 2A,B).

### 2.3. Expression of BK_Ca_ Channels in NHEKs

**Figure 3:**
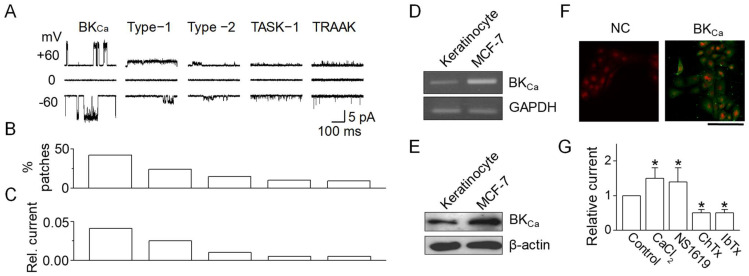
Expression of BK_ca_ channels in human keratinocytes. (**A**) Single-channel currents in cell-attached patches of NHEKs. Recordings from cell-attached patches on NHEKs revealed five types of channels, each with distinct opening kinetics. Both pipette and bath solutions contained 150 mM KCl. The K+ selectivity of each channel type was confirmed by measuring reversal potential shifts after altering the bath (KCl) and by the absence of outward current in Na^+^-containing solutions. (**B**) Distribution of K^+^ channel types. The bar graph shows the percentage of patches exhibiting each type of K^+^ channel identified in the NHEKs. (**C**) Estimated average relative outward current, compiled from 125 patches. (**D**) RT-PCR analysis for BK_Ca_ channel in NHEKs. MCF-7 cells were used as a positive control. (**E**) Western blotting for BK_Ca_ channels in NHEKs. (**F**) Localization of BK_Ca_ in NHEKs. Scale bar, 100 μm. (**G**) Pharmacological characterization of BK_Ca_ channels in NHEKs. The data are presented as mean ± SD from five independent experiments. * *p* < 0.05 compared to control.

To determine the involvement of specific K^+^ channels in wound healing processes, the patch clamp technique was utilized. This study excluded voltage-dependent K^+^ channels as potential contributors to NHEK wound healing due to the lack of effect from Ba^2+^. Initially, single-channel recordings were conducted to ascertain the functional presence of K^+^ channels. Cell-attached patches were created, recording all channel activities at both −60 and +60 mV in solutions containing 150 mM KCl for both bath and pipette. Among the 125 patches examined, five distinct background K^+^ channels were identified, which were differentiated by their unique single-channel conductance and kinetic properties (Figure 3A). This study further delved into ion selectivity, pharmacological characteristics, and kinetics for each channel type. A BK_Ca_-like channel was identified due to its sensitivity to CaCl_2_, frequently observed in cell-attached patches (42% of patches, Figure 3B). This channel exhibited single-channel conductances of 174 ± 15 pS at +60 mV and 166 ± 14 pS at −60 mV (*n* = 5), forming a linear current–voltage relationship characteristic of BK_Ca_ channels. Compared to other K^+^ channels, BK_Ca_-like channel activity was relatively prominent (Figure 3C). In addition, TWIK-related acid sensitive potassium (TASK)-1- and TWIKrelated arachidonic acid-stimulated potassium (TRAAK)-like channels, belonging to the two-pore domain K^+^ (K_2P_) channel family, were observed in keratinocytes. These channels, with conductances of 15-pS and 73-pS, are shown in Figure 3A. TASK-1- and TRAAK-like channels were activated by alkalization of the extracellular and intracellular environments, respectively. However, due to their low levels in NHEKs, K_2P_ channels (TASK-1- and TRAAK-like) were not considered significant contributors.

To confirm BK_Ca_ expression in NHEKs, RT-PCR, Western blotting, and immunocytochemistry were employed. RT-PCR results revealed the presence of BK_Ca_ mRNA in NHEKs (Figure 3D). Western blot analysis aligned with RT-PCR findings, indicating BK_Ca_ protein expression in NHEKs, evidenced by a ~140 kDa band, similar to that observed in MCF-7 cells known to express BK_Ca_ channels (used as a positive control) (Figure 3E). BK_Ca_ channels were localized in various cellular regions, including perinuclear areas, intracellular compartments, and the cell membrane, but not in the nucleus (Figure 3F). The characteristic features of BK_Ca_ channels, such as activation by CaCl_2_ and NS1619 and inhibition by charybdotoxin (ChTx) and iberiotoxin (IbTx), were also investigated. These properties were assessed in symmetrical 150 mM K^+^ solutions in both the pipette and bath. As shown in Figure 3G, BK_Ca_-like channel activity significantly increased following patch excision with 5 μM CaCl_2_ application. In outside-out patches, the addition of 30 µM NS1619 to the bath solution notably enhanced channel activity, while 100 nM ChTx and 100 nM IbTx effectively reduced the BK_Ca_ current.

### 2.4. Involvement of BK_Ca_ Channels in Wound Healing

**Figure 4:**
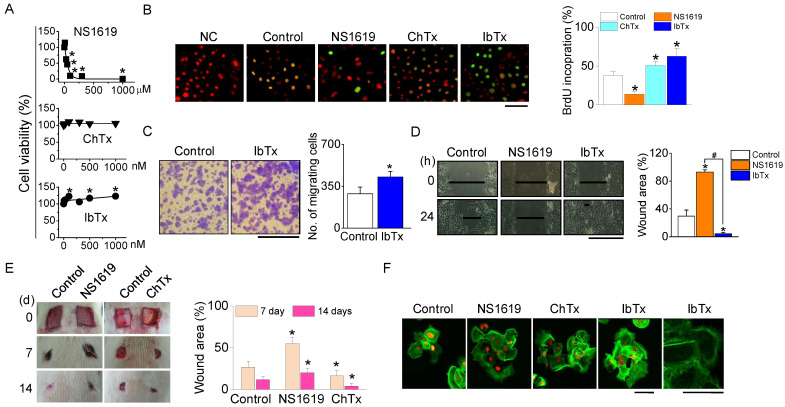
Effect of BK_Ca_ channel modulators on NHEKs proliferation and migration. (**A**) Cytotoxicity of BK_Ca_ channel activator. Dose-response curve for cell viability following a 24 h exposure to the NS1619, ChTx, and IbTx. (**B**) Effect of BK_Ca_ channel modulators on NHEK proliferation. NHEKs were treated with NS1619 (30 µM), ChTx (300 nM), and IbTx (300 nM). The proliferation rate was assessed by counting the number of BrdU-positive cells and PI-stained cells within a 200 µm radius from the wound edge 12 h post-wounding (*n* = 4). Scale bar, 50 µm. (**C**) Effect of IbTx on NHEK migration. Representative microscopic images were captured 12 h after treatment with IbTx (*n* = 3). Scale bar, 100 µm. (**D**) In vitro wound healing assay. Light micrographs depict the wound healing process in NHEK monolayers (*n* = 4). Scale bar, 500 µm. (**E**) In vivo wound healing assay after treatment of BK_Ca_ channel modulators (*n* = 4). (**F**) Filopodia formation. Staining with Oregon Green^®^ 488 phalloidin was used to visualize filopodia (*n* = 5). Scale bars, 40 µm. The data are presented as mean ± SD from four or five independent experiments. * *p* < 0.05 compared to each corresponding control. ^#^ *p* < 0.05 compared between NS1619 and IbTX treatment.

To elucidate the role of BK_Ca_ channels in the migration and proliferation of NHEKs during the wound healing process, we first assessed the cytotoxicity of BK_Ca_ channel modulators (NS1619, ChTx, and IbTx) on NHEKs. While IbTx and ChTx, at concentrations ranging from 0 to 1000 nM, did not induce significant cell death, NS1619 was found to be cytotoxic, causing 50% cell death at a concentration of 55 µM (Figure 4A). The wound healing process involves the repopulation of the wounded area by adjacent cells through proliferation and migration. To differentiate these processes, we separately evaluated cell migration and proliferation. Furthermore, 5-bromo-2′-deoxyuridine (BrdU) incorporation assays were used to measure cell proliferation during wound closure in NHEK cultures. A higher number of BrdU-positive cells were observed in the cultures treated with ChTx and IbTx (Figure 4B). In transwell migration assays, the migration of IbTx-treated cells towards a 10% FBS gradient was significantly higher compared to control cells (*p* < 0.05, Figure 4C). IbTx exhibited effects similar to ChTx in wounded NHEKs.

For in vitro wound healing assays, we created a ~600 µm wide defect in confluent NHEK monolayers by scratching. The addition of IbTx dramatically accelerated wound closure in NHEKs, whereas NS1619 exhibited an inhibitory effect on wound healing 24 h post-wound creation (Figure 4D). Correspondingly, in vivo wound healing assays demonstrated that both ChTx and IbTx facilitated wound closure, while NS1619 hindered this process (Figure 4E).

To further understand the influence of BK_Ca_ channel modulators on cellular structure, we examined the actin cytoskeleton in NHEKs stained with phalloidin. Cells treated with ChTx and IbTx displayed more extensive filopodia formation compared to the control group. In contrast, although polymerized actin was present in NS1619-treated cells, these cells exhibited a reduced area covered by polymerized actin and with fewer filopodia, and the existing filopodia were shorter and less organized (Figure 4F).

### 2.5. Enhanced Wound Healing in BK_Ca_ Channel-Deficient Keratinocytes

**Figure 5:**
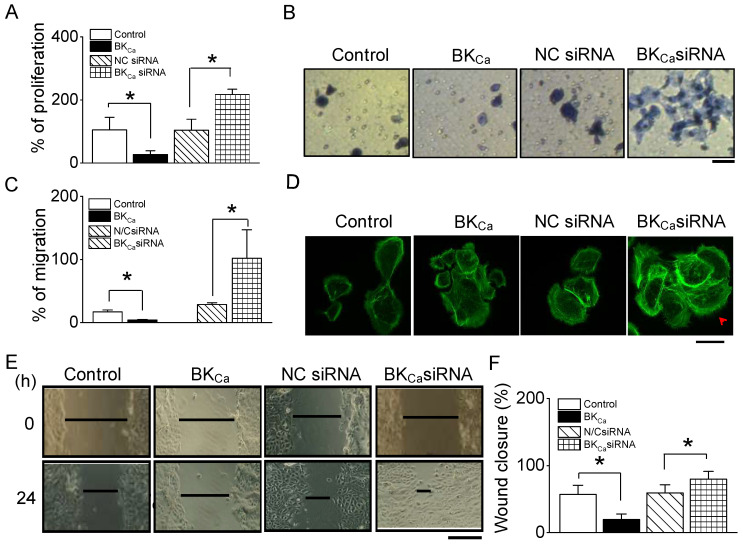
Wound healing facilitated by BK_Ca_ siRNA. (**A**) Increase in proliferation by BK_Ca_ siRNA. Summary of the percentage change in cell proliferation of NHEKs with manipulated BK channels (*n* = 5). (**B**) Enhanced migration of BK_Ca_ siRNA-transfected cells. Scale bar, 100 µm. (**C**) Summary of cell migration changes by overexpression and knock-down of BK_Ca_ channels (*n* = 5). (**D**) Morphological actin changes in BK_Ca_ knocked down NHEKs. The arrow head indicates filopodia extruded from NHEKs. Scale bar, 20 µm. (**E**) Accelerated wound healing in BK_Ca_ knocked down NHEKs. Scale bar, 300 µm. (**F**) Summary of wound closure changes by overexpression and knock-down of BK_Ca_ channels (*n* = 5). Control represents vector-transfected cells. NC represents negative control. The data are presented as mean ± SD from five independent experiments. * *p* < 0.05 compared to each corresponding control.

CWH was facilitated by the inhibition of BK_Ca_ channels (Figure 4). Building on this observation, we aimed to determine if the knockdown of endogenous BK_Ca_ channels would similarly facilitate CWH. To test this, we conducted experiments using NHEKs transfected with human BK_Ca_-plasmid DNA and compared their proliferation and migration capabilities to cells transfected with an empty vector. NHEKs with BK_Ca_ overexpression exhibited reduced proliferation and migration. Conversely, NHEKs transfected with siRNA targeting BK_Ca_ demonstrated a significant increase in both proliferation and migration compared to cells transfected with negative control (NC) siRNA, which consisted of a scrambled sequence (*p* < 0.05, Figure 5A,B). In addition, the migration assay revealed that BK_Ca_ siRNA-transfected cells exhibited approximately a four-fold increase in cell migration compared to NCsiRNA-transfected cells (Figure 5C). Morphological analysis of these cells showed that NHEKs with BK_Ca_ knockdown had an increased number of filopodia, whereas cells overexpressing BK_Ca_ displayed fewer filopodia (Figure 5D). The effect of BK_Ca_ channel modulation was further confirmed in wound healing assays. These assays demonstrated a decreased rate of wound closure in cells overexpressing BK_Ca_ channels while silencing BK_Ca_ expression led to significantly enhanced wound closure compared to their corresponding controls (Figure 5E,F).

## 3. Discussion

We have shown that inhibiting BK_Ca_ channels enhances CWH through the modulation of cell proliferation, migration, and actin filament dynamics. To investigate the role of K^+^ channels in this process, we conducted wound healing experiments using high concentrations of potassium chloride (KCl). Particularly, we used pharmacologically high concentrations (25 mM) of KCl to act as a K^+^ channel blocker in NHEKs. Considering its value as a wound healing agent, high concentrations of KCl may be more economical than specific channel blockers and have a wider range of applications. Furthermore, the efficacy of K^+^ channel blockade in promoting wound healing was also validated in both murine and porcine models, underscoring the broad relevance of K^+^ channels in the wound healing process across different species.

### 3.1. BK_Ca_ Channel as a Target of Wound Healing

Various types of Ca^2+^-activated K^+^ (K_Ca_) channels are known to be expressed in human keratinocytes. These include BK_Ca_ channels in oral keratinocytes and HaCaT cells [16,17], the intermediate conductance Ca^2+^-activated K^+^ channel (hIK1) in HaCaT cells and epidermal keratinocytes [14,18], and the small conductance Ca^2+^-activated K^+^ channel type 4 (SK4) in HaCaT cells [19]. However, there are few reports on the expression of BK_Ca_ channels in NHEKs rather than cell lines. In the present study, we have successfully identified the expression of BK_Ca_ channels in NHEKs. The BK_Ca_ channel in NHEKs can be distinctly isolated from other K^+^ channels due to its unique response to specific modulators. This channel is characterized by its high unitary conductance and sensitivity to Ca^2+^. BK_Ca_ channels are inhibited by charybdotoxin (ChTx), iberiotoxin (IbTx), and low concentrations of tetraethylammonium (TEA), and they are activated by NS-1619 [20,21].

K_Ca_ channels are known to be expressed in keratinocytes, but their functional role in these cells remains largely unexplored. In contrast, the function of BK_Ca_ channels in other cell types has been extensively studied, particularly in relation to cell proliferation, migration, and metastasis. Opening BK_Ca_ channels contributes to the migratory ability of glioblastoma stem-like cells [22], enhances the colonization of hepatocellular carcinoma cells [23], and induces cell death in triple-negative breast cancer [24]. BK_Ca_ activity is crucial for controlling cell proliferation by modulating the cell membrane potential in MCF-7 cells [25]. In addition, the expression levels of BK_Ca_ channels have been linked to the migration and invasion capabilities of human glioma cells [26]. NS1619, a BK_Ca_ channel activator, has been shown to exert an anti-proliferative effect in A2780 ovarian cancer cells, which is associated with increased expression of p53, p21, and Bax [27]. The effect of BK_Ca_ channels on cell migration is also evident, as demonstrated by the reduced migration observed in transformed renal epithelial cells in the presence of ChTx [28]. Interestingly, ChTx has been reported to promote wound healing in human colon carcinoma T84 monolayers [11], while another study found that ChTx had no effect on myoblast migration [29].

Most studies on the role of BK_Ca_ channels have been focused on cancer cells, where BK_Ca_ channel modulators have been observed to influence the proliferation and migration of these cells, albeit with varying effects across different cell types. This variability is likely attributable to differences in the distribution and proportion of BK_Ca_ channels expressed in each type of cell. In contrast, the proliferation and migration of normal cells have been less extensively studied. Nevertheless, the findings from previous studies imply that BK_Ca_ channels play a significant role in the proliferation and migration of mammalian cells, including normal cells.

Keratinocytes, which form the predominant cell population in the skin, play a crucial role in CWH through the modulation of their proliferation and migration in conjunction with fibroblasts. In the present study, we identified the presence of BK_Ca_ channels in keratinocytes, suggesting that these channels may be involved in the processes of cell proliferation and migration initiated during wound healing. In addition, BK_Ca_ channels have been reported to be expressed in human dermal fibroblasts [30]. The concurrent expression of BK_Ca_ channels in both keratinocytes and fibroblasts could potentially enhance the synergistic function of these channels in CWH.

In the present study, we observed that the downregulation of BK_Ca_ channel expression facilitated CWH, whereas the upregulation of BK_Ca_ channel expression delayed the healing process. Beyond channel expression, the modulation of K^+^ channel activity is known to impact cell proliferation, apoptosis, and migration [31,32]. Various K^+^ channel blockers have been shown to inhibit cell migration and proliferation in cells derived from different organs [33,34]. Conversely, the inhibition of K^+^ channels can accelerate wound healing in intestinal epithelial cells, which is associated with the co-regulation of F-actin polymerization and volume control [11]. We found that BK_Ca_ channel blockers expedite keratinocyte wound healing by co-regulating cell proliferation, migration, and F-actin polymerization.

Cell volume and actin polymerization are closely interconnected during the process of cell migration. The efflux of K^+^ ions and/or the expression of K^+^ channels play crucial roles in determining the balance between cell survival and death across various cell types. The plasma membrane K^+^ conductance is a key factor in both volume regulation and actin polymerization [11]. In keratinocytes, BK_Ca_ channel activity contributes to the efflux of K^+^ across the cell membrane, which is a vital aspect of cellular function. In a wide range of cells, volume regulation through water loss is often linked with Cl^−^ secretion, a process that typically involves the activation of K^+^ channels [35]. This K^+^ conductance tends to function relatively early and for a brief duration in the healing process of intestinal epithelial cells. Inhibition of K^+^ channels can lead to transient alterations in wound repair mechanisms [11]. Such inhibition may help to prevent volume shrinkage, thereby averting a later stage of apoptosis. Consequently, the blockage of K^+^ efflux by K^+^ channel blockers can act as a protective mechanism against apoptosis [36]. This intricate relationship between K^+^ channel activity, cell volume regulation, and actin polymerization underscores the complexity of cellular responses during processes like wound healing. Understanding these mechanisms can provide valuable insights into the development of therapeutic strategies for various pathological conditions.

### 3.2. Potential Role of BK_Ca_ Channel Blockers in Cutaneous Wound Healing

Therapeutic agents used in wound treatment should ideally promote healing steps effectively while minimizing harmful side effects [37]. EGF, recognized as a prominent promoter of wound healing, plays a multifaceted role in the wound healing process. Its functions encompass a range of activities including cellular proliferation, wound contraction, collagen deposition, and changes in both the morphology and migration of keratinocytes [38]. Similar to EGF, but potentially differing in their mechanisms of action, agents like 25 mM KCl, ChTx, and IbTx have also been demonstrated to positively influence wound healing. Their effects are primarily seen in the promotion of cell proliferation, migration, and the polymerization of F-actin, all achieved without inducing cytotoxicity. In the present study, we observed that a 25 mM concentration of KCl enhances the wound healing process in both rat and pig models. A reduced erythema ratio observed with the application of 25 mM KCl indicates progress in wound healing stages. Assessing the erythema ratio, which measures the redness in the wound area, is useful for understanding the healing stage, evaluating inflammation levels, and identifying potential complications such as infection. Furthermore, in our previous study, we observed a significant increase in the protein level of vascular endothelial growth factor (VEGF) during the first week of wound healing in wounds treated with 25 mM KCl [39]. VEGF enhances wound healing through various mechanisms such as collagen deposition, angiogenesis, epithelization, and fibroblast migration [40,41]. Given the anatomical and physiological similarities between pig skin and human skin [42], it is advisable to conduct experiments on pig skin before applying various chemicals to human wounds. This approach ensures that chemicals demonstrating positive effects on porcine skin can be more confidently considered for clinical trials.

### 3.3. Potential Limitation

To investigate the specific K^+^ channels involved in wound healing, we employed a methodology that involved applying high concentrations of KCl to assess their impact. This effect was further substantiated using a range of K^+^ channel blockers. Through patch-clamp experiments, we identified a predominantly expressed subtype of the K^+^ channel in the cells, which was determined to be the BK_Ca_ channel. Despite this significant finding, there are still unresolved questions regarding the BK_Ca_ channels. In theory, treating cells with high concentrations of KCl should lead to an increase in intracellular Ca^2+^ levels, potentially activating the BK_Ca_ channels. However, in our experiments, the application of 25 mM KCl to BK_Ca_ channels did not result in any noticeable activation of these channels. This unexpected outcome suggests that the relationship between KCl treatment and BK_Ca_ channel activation is not straightforward and warrants further investigation to fully understand the underlying mechanisms.

In addition, this study raises the possibility that, in addition to the BK_Ca_ channels identified in keratinocytes, TWIK-related potassium (TREK)-2 channels may also be present. TREK-2 channels are occasionally misidentified as BK_Ca_ channels due to their broad range of conductance magnitudes [43]. Interestingly, TREK-2 channels are regulated by extracellular Ca^2+^. The characteristics of large conductance TREK-2 channels are not well understood, and this area merits further exploration. This is particularly important considering that TREK-2 channels have the capability to form heterodimers with other members of the same family, such as TREK-1 and TRAAK [44]. The potential presence and functional implications of TREK-2 channels in keratinocytes, especially in the context of their interaction with other channel types, could provide valuable insights into the complex mechanisms of cellular signaling and regulation.

The in vivo wound model applied in our study did not utilize a splint for stabilizing the wound area. Splinting is often employed in animal models, such as mice and rats, to inhibit wound contraction [45]. In contrast, human wound closure relies more heavily on re-epithelialization, i.e., the regrowth of skin cells. Animal studies frequently use splint models to better replicate human wound healing dynamics. While our non-splint model still demonstrated re-epithelialization, future studies should employ a splint model to more accurately investigate the role of the BK_Ca_ channel in this process.

From this study, we propose that high concentrations of KCl, as well as BK_Ca_ blockers, could serve as effective enhancers for human CWH. Notably, there has been a reported trial for healing donor sites of split-thickness skin grafts using KCl, where a 1 M potassium/calcium chloride solution in hydrogen was employed [46]. This approach underscores the potential therapeutic applications of KCl in wound healing. Our study offers new insights into the function of BK_Ca_ channels and high concentrations of KCl in CWH. The involvement of BK_Ca_ channels in cell proliferation and migration is particularly significant as it may provide crucial information about the role of BK_Ca_-expressing cells in various tissues, including the skin. Understanding these mechanisms is likely to enhance our comprehension of the physiological and pathophysiological processes involved in wound healing.

## 4. Materials and Methods

### 4.1. Chemicals

All chemicals used in this study were sourced from Sigma Chemical Co. (St Louis, MO, USA), except where noted. Stock solutions were prepared in dimethyl sulfoxide (DMSO) for charybdotoxin (ChTx, 100 µM), iberiotoxin (IbTx, 23.6 µM), and NS1619 (50 mM). Epidermal growth factor (EGF, 10 mM) and potassium chloride (KCl, 1 M) were dissolved in distilled water. These stock solutions were then diluted to working concentrations using a culture medium or phosphate-buffered saline (PBS). For experiments involving DMSO, a control solution with an equivalent DMSO concentration was used. The final DMSO concentrations were approximately 0.1% (*v*/*v*) for all chemicals, except for ChTx and IbTx, where they were about 0.3% and 1.3% (*v*/*v*), respectively.

### 4.2. Animals

Sprague Dawley rats (male, 250 g, *n* = 13) and Yorkshire pigs (2 months old, 20 kg) were acquired from Koatech Co. (Animal Breeding Center, Pyeongtaek, Republic of Korea) and XP-bio Inc. (Ansung, Republic of Korea), respectively. The animals were housed in a pathogen-free environment under a 12 h light/dark cycle, with ad libitum access to food and water. They were kept in separate cages, with the temperature controlled between 23 and 26 °C and humidity maintained at around 65%. All animal experiments were conducted in strict accordance with the guidelines for animal research set forth by the National Institutes of Health. The protocols for these experiments were reviewed and approved by the Institutional Animal Care and Use Committee at Gyeongsang National University and Seoul National University (Approval Numbers: GLA-090805-R0065, IACUC No. 09-0097, and 09-0088).

### 4.3. Cell Culture

Normal human epidermal keratinocytes (NHEKs) from an 11-year-old donor (passage 1) were obtained from Welskin (Seoul, Republic of Korea). The NHEKs were cultured in keratinocyte growth medium (KBM^®^, Cambrex Bioscience, Walkersville, MD, USA), supplemented with bovine pituitary extract (0.4% *v*/*v*), human recombinant EGF (0.125 ng/mL), insulin (5 μg/mL), hydrocortisone (0.33 μg/mL), transferrin (10 μg/mL), epinephrine (0.39 μg/mL), CaCl_2_ (0.15 mM), amphotericin B (50 ng/mL), and gentamicin (50 μg/mL), following the manufacturer’s recommendations. Human neonatal dermal fibroblasts (HNDFs) were purchased from Cambrex Bio Science (Walkersville, MD, USA). Both HaCaT cells and HNDFs were maintained in DMEM (Gibco BRL, Grand Island, NY, USA) supplemented with 10% heat-inactivated fetal bovine serum (FBS) (Hyclone, Logan, UT, USA), 100 U/mL penicillin, and 100 µg/mL streptomycin. The cells were incubated at 37 °C in a humidified atmosphere containing a 95% air–5% CO_2_ gas mixture. The culture medium was replaced every two days. For electrophysiological experiments, NHEKs (passages 1–3) were seeded onto poly-L-lysine-coated glass coverslips and used within 3 days of seeding.

### 4.4. Cell Viability Assay

Cell viability was assessed colorimetrically using the 3-(4,5-dimethylthiazole-2-yl)-2,5-diphenyl tetrazolium bromide (MTT) assay. Cells in their exponential growth phase were seeded at a density of 5 × 10^3^ cells per well in 96-well plates. After a 24 h treatment with the indicated chemicals, 10 µL of MTT solution (5 mg/mL) was added to each well, followed by an incubation period of 4 h. Subsequently, the supernatants were carefully removed, and the formazan crystals formed in each well were dissolved in 100 µL of DMSO for 30 min at room temperature. The absorbance values for each well were then measured using a Microplate Reader (Bio-Rad, Hercules, CA, USA) to determine the relative cell viability.

### 4.5. Proliferation Assay

Cell proliferation was assessed using the 5-bromo-2’-deoxyuridine (BrdU) incorporation method. Briefly, NHEKs were incubated at 37 °C for 2 h with 10 µM BrdU. Following the incubation, the cells were fixed in 4% paraformaldehyde. Subsequently, they were stained with a FITC-conjugated anti-BrdU monoclonal antibody to detect the incorporated BrdU, which is indicative of DNA synthesis and thus cell proliferation. To quantify proliferation, BrdU-positive cells were counted within a 200 μm distance from the wound margin. The results were then expressed as a percentage of the total number of BrdU-positive cells, providing a measure of the proliferative activity of the cells in response to the treatment. Additionally, total cellular DNA was quantified using propidium iodide (PI) staining.

### 4.6. Cell Migration Assays

The transwell migration assay was conducted using the CytoSelect™ Cell Migration Assay Kit, which includes polycarbonate membrane inserts with 8 μm pores (Cell Biolabs, Inc., San Diego, CA, USA), following the manufacturer’s instructions. Initially, a cell suspension containing 1 × 10^5^ cells/mL in a growth factor-free medium was prepared. Then, 300 µL of this suspension, with BK_Ca_ channel modulators added directly to it, was placed in the upper chamber of each insert. The lower chamber was filled with a medium containing 10% FBS to facilitate cell migration towards the lower face of the transwell culture inserts. The cells were incubated for 6 h at 37 °C in a humidified atmosphere of 95% air and 5% CO_2_. After incubation, non-migrating cells on the inner side of the transwell culture inserts were gently removed using a cotton-tipped swab. The cells that had migrated to the bottom surface were stained with a cell-staining solution (0.5% cresyl violet or methylene blue) for 10 min at room temperature. Photomicrographs of five individual fields per insert were captured using a microscope (Axiovert 40C, Carl Zeiss MicroImaging, Göttingen, Germany). The number of migrated cells in these fields was counted to calculate the average migration. This assay was also performed with cells that had overexpressed or knocked down BK_Ca_ channels, following the same protocol as for the chemical-treated cells. To confirm the results of the transwell migration assay, cell monolayers grown to confluence in a µ-dish (ibidi GmbH, Munich, Germany) were treated with 8 µg/mL mitomycin C in culture media for 2 h to inhibit cell proliferation. This step ensured that the observed cell migration was not influenced by cell division.

### 4.7. Collagen Assays

A collagen assay (Sircol Collagen Assay Kit, Biocolor, UK) was employed to measure collagen concentration. Human neonatal dermal fibroblasts (HNDFs) were first seeded in a 24-well plate and treated with different concentrations of KCl in DMEM containing 2% FBS. After 72 h, the media was collected from each well for collagen concentration analysis. For the assay, 450 μL of the collected media was added to 1.5 mL micro-centrifuge tubes containing 300 μL of Sircol Dye reagent. The mixture in each tube was then shaken mechanically for 30 min (CR100, Finemould Precision Ind. Co., Seoul, Republic of Korea) and centrifuged at 1000× *g* for 10 min. The supernatant was carefully removed, and the dye-bound collagen pellets were dissolved in 300 μL of alkali reagent. Aliquots of 200 μL were transferred from the micro-centrifuge tubes to a 96-well plate, and the absorbance was measured using a Microwell Plate Colorimeter (VersaMax; Molecular Devices, Sunnyvale, CA, USA) at a wavelength of 540 nm. This process enabled the quantification of collagen produced and secreted by the HNDFs under the influence of 25 mM KCl.

### 4.8. Reverse Transcriptase (RT)—Polymerase Chain Reaction (PCR) Analysis

First-strand cDNAs were synthesized from total RNA isolated from NHEKs using oligo dT primers, as part of the SuperScript™ First-Strand Synthesis System for RT-PCR (Invitrogen, Carlsbad, CA, USA). This synthesized first-strand cDNA served as the template for subsequent PCR amplification. For PCR, specific primers targeting the BK_Ca_ channel (GenBank accession number NM_001014797) were used: sense primer 5′-TAATTCCCAAGGGTTCACACC-3′ and antisense primer 5′-GCTTTGCAGAACAGATCACCA-3′. These primers were designed to span exons, thereby helping to identify any genomic DNA contamination and amplify short DNA fragments specific to the BK_Ca_ channel in NHEKs. The PCR amplification was performed using Taq polymerase (Takara, Otsu, Japan) under the following conditions: initial denaturation at 94 °C for 5 min, followed by 30 cycles of 94 °C for 30 s, 55 °C for 45 s, and 72 °C for 45 s, and a final extension step at 72 °C for 10 min. The DNA fragment amplified from NHEKs via RT-PCR was then directly sequenced using the ABI PRISM^®^ 3100-Avant Genetic Analyzer (Applied Biosystems, Foster City, CA, USA). This sequencing process allowed for the verification of the specific BK_Ca_ channel gene expression in NHEKs.

### 4.9. Western Blot Analysis

NHEKs were lysed using a protein extraction solution (PRO-PREPTM, iNtRON Biotechnology Inc., Seongnam, Republic of Korea) containing a mixture of 50 mM Tris-Cl (pH 7.5), 150 mM NaCl, 1 mM dithiothreitol (DTT), 0.5% NP-40, 1% Triton X-100, 1% deoxycholate, 0.1% SDS, 1 mM EDTA, 1 µM leupeptin, 1 µM pepstatin A, 1 mM phenylmethylsulfonyl fluoride (PMSF), and 0.1 µM aprotinin. The lysates were incubated on ice for 60 min with intermittent vortexing. The extracts were then clarified by centrifugation at 13,000 rpm (16,609× *g*, Micro 17TR, Hanil, Republic of Korea) for 20 min at 4 °C. The supernatants were collected post-centrifugation, and the total protein content was quantified using the Bradford protein assay (Bio-Rad, Hercules, CA, USA). For Western blot analysis, 30–50 μg/lane of total proteins were separated on 10% SDS-polyacrylamide gels and transferred onto polyvinylidene fluoride (PVDF) membranes (0.45 μm, Millipore, Bedford, MA, USA) in a transfer buffer (TBS; 25 mM Tris-base, 190 mM glycine, and 20% methanol) using a semi-dry blotter (Bio-Rad). The membranes were blocked with 5% fat-free milk and 0.05% Tween20 in TBS for 1 h, followed by overnight incubation at 4 °C with goat anti-MaxiKα (BK_Ca_) polyclonal antibody (Santa Cruz Biotechnology, Inc., Santa Cruz, CA, USA) at a 1:500 dilution. Horseradish peroxidase (HRP)-conjugated rabbit anti-goat secondary antibody (1:3000; assay designs, Ann Arbor, MI, USA) was applied at room temperature for 1 h. Antigens were detected using enhanced chemiluminescence (ECL Plus kit; ELPIS, Taejeon, Republic of Korea) as per the manufacturer’s instructions.

### 4.10. Immunocytochemistry and Actin Staining

NHEKs cultured on round coverslips coated with poly-L-lysine were processed for immunofluorescence as follows. The cells were first washed in PBS and then fixed with 4% paraformaldehyde in 0.1 M PBS for 30 min. After washing in PBS, the cells were permeabilized with 0.1% Triton X-100 (Fisher Scientific, Pittsburgh, PA, USA) in PBS for 10 min. They were then preincubated in a blocking buffer containing 1% normal rabbit serum (NRS) for 2 h at room temperature with gentle rotation. The cells were incubated overnight at 4 °C with an affinity-purified polyclonal antibody against BK_Ca_ protein, diluted 1:200 in PBS containing 0.1% (*w*/*v*) gelatin, 0.05% (*w*/*v*) NaN_3_, and 1% (*v*/*v*) NRS. Following three washes in PBS, the cells were incubated for 1.5 h in the dark with FITC-conjugated rabbit anti-goat IgG, which was diluted 1:400 in PBS. Subsequently, the cells underwent PI staining for nuclei visualization. The coverslips with cultured cells were then rinsed and wet-mounted on glass slides using Gel/Mount™ reagent (Biomeda Corp., Foster City, CA, USA). Negative and positive controls were included, with the former being achieved by omitting the primary antibody and the latter by transfecting BK_Ca_ plasmid DNA. A competition control was also performed, which was pre-absorbed with a 2-fold excess of antigenic peptide. In the negative and competition controls, no green fluorescence was observed, only the PI stain was visible. The stained cells were examined using a confocal laser scanning microscope equipped with a ×40 objective (CLSM, IX70 Fluoview, Olympus, Tokyo, Japan). For F-actin staining, NHEKs fixed with 4% paraformaldehyde were treated with Oregon Green^®^ 488 phalloidin (1:50, Molecular Probes, Eugene, OR, USA) for 30 min at room temperature. After staining, the coverslips were rinsed three times with PBS, mounted on slides, and imaged on the same day.

### 4.11. In Vitro Wound Healing Assays

For the wound healing assays, cells were seeded in a 24-well culture dish at a density of 1×10^5^ cells per well. Once the cells reached confluence, a sterile pipette tip was used to create a scratch wound across the cell monolayer. The cells were then washed with PBS to remove detached cells and debris, and a fresh medium containing various K^+^ channel modulators was added to each well. Microphotographs of the scratched area were taken after incubation for specified time periods. To quantify wound closure, the width of the wound in the monolayer was measured under a microscope (Axiovert 40C). Relative wound closure was calculated by subtracting the measured wound width at each time point from the initial wound width (measured at 0 h). This difference was then expressed as a percentage of the initial wound width, providing a quantitative measure of wound closure over time. The measurements were taken from three separate scratches in each well to ensure data reliability and accuracy.

### 4.12. In Vivo Wound Healing

The wound model was developed using a modified version of the protocol described in previous studies [47,48,49]. These models demonstrate wound re-epithelialization through histological analysis [49,50]. Rats were anesthetized with an intraperitoneal injection of sodium pentobarbital (45 mg/kg). The skin on their backs was shaved using a safety razor, and two rectangular-shaped wounds, each measuring either 2 × 2 cm or 1.5 × 1.5 cm, were created. Following skin sterilization, full-thickness wounds, including both the epidermis and dermis, were excised using a number 10-blade scalpel. Any bleeding points were coagulated. KCl (25 mM) and BK_Ca_ modulators were applied daily to all right-sided wounds (experimental group), while an equivalent volume of PBS was applied to all left-sided wounds (control group). The wound areas on the rats were monitored for 21 days post-wounding, with photographs taken on days 0, 7, 14, and 21. The healing ratio was calculated by comparing the measured wound dimensions to the original wound dimensions on each day. For the pig model, sedation was achieved with an intramuscular injection of 2.2 mg/kg azaperone (Stresnil^®^, Janssen, Belgium), followed by an injection of 3 mg/kg propofol (Diprivan-PFS^®^, AstraZeneca Korea, Republic of Korea) into the external ear vein for anesthesia induction. The hair on the pigs’ backs was removed, and a 5 × 5 cm full-thickness wound was created using a dermatome (Acculan^®^, Aesculap Inc., Center Valley, PA, USA) set to a depth of 18/1000 inch. Post-wounding, Aquacel^®^ (Convatec Professional Services, Bridgewater, NJ, USA) was soaked with 25 mM KCl for the experimental group and PBS for the control group. The Aquacel was then applied to each wound, and an Ioban^®^ (3M Corporate, St. Paul, MN, USA) dressing was used to cover the Aquacel for 2 days. Additionally, a bandage was applied to protect the dressing. Wound dimensions and erythema were measured at 5 and 10 days postoperatively. The healing ratios were calculated based on the measured wound dimensions compared to the original wound dimensions at postoperative day 5, and the erythema ratios were calculated based on the measured erythema dimensions compared to the original wound dimensions at postoperative day 10.

### 4.13. H&E and Masson’s Trichrome Stains

For histological analysis, rat wound tissues (at a 5 mm border surrounding the injuries) were harvested from both the control and experimental groups two weeks after surgery. The skin samples were washed in 0.1 M PBS and fixed with 4% (*w*/*v*) paraformaldehyde in 0.1 M PBS. They were then processed and embedded in paraffin, and sections of 4 μm thickness were cut. These sections were air-dried on gelatin-coated slides, deparaffinized, and washed with tap water for 5 min. The sections were stained with hematoxylin for 5 min, with staining completeness checked in tap water. Eosin staining was subsequently performed for 3 min. The sections were dehydrated through a graded series of alcohols (70 to 100% ethanol, 3 min each), cleared in xylene, and mounted with coverslips. Stained sections were photographed using a microscope (BX-51, Olympus, Tokyo, Japan) equipped with a high-resolution video camera (Camedia C-7070, Olympus). Five sections from each sample were evaluated for histological changes.

For Masson’s trichrome staining, the sections were first deparaffinized with alcohol, and then stained in Weigert’s iron hematoxylin working solution for 10 min. After rinsing under running warm tap water for 10 min, they were stained in Biebrich scarlet-acid fuchsin solution for 15 min. The sections were differentiated in a phosphomolybdic–phosphotungstic acid solution for 15 min and then transferred directly to an aniline blue solution for 5–10 min of staining. Following dehydration through 95% and 100% ethanols, the sections were mounted with a resinous mounting medium. This staining method highlights collagen fibers in blue, providing a clear contrast to other tissue components and aiding in the assessment of tissue repair and fibrosis.

### 4.14. Transfection

The human BK_Ca_ channel clone was kindly provided by Prof. C. S. Park (Gwangju Institute of Science and Technology, Gwangju, Republic of Korea). NHEKs were plated at 1 × 10^5^ cells per dish in 24-well plates, 24 h before transfection, in KBM supplemented with growth factors. For overexpression or silencing of BK_Ca_, NHEKs underwent transfection with either BK_Ca_ DNA in pcDNA3.1 or BK_Ca_-specific siRNA, utilizing the Magnetofection™ system. A mixture of 1.0 µg of DNA and 1.0 µL of PolyMAG (Chemicell GmbH, Berlin, Germany) was prepared and incubated for 20 min at room temperature. This mixture was then added to 500 µL of growth factor-depleted culture medium in each well. The plates were positioned on a MagnetoFACTOR plate 24 device for 20 min at room temperature. After this, the culture medium was replaced with fresh medium, and the cells were incubated at 37 °C in a 95% air and 5% CO_2_ atmosphere. The expression of the transgene was assessed two days post-transfection.

### 4.15. Gene Silencing with siRNA of Human BK_Ca_ Channels

Human BK_Ca_ channel-specific oligonucleotides were synthesized using Invitrogen’s Stealth™ siRNA technology. NHEKs were transfected with either 80 pM of a negative control siRNA or BK_Ca_-specific siRNA sequences (5′-UUUGAGUGAUGAUUCUUAUCUUCGG-3′ and 5′-CCGAAGAUAAGAAUCAUCAUCCAAA-3′). This transfection was performed in a growth factor-free medium using the Magnetofection™ system. As a positive control for transfection efficiency, siGLO Lamin A/C siRNAs (Dharmacon^TM^, Lafayette, CO, USA) were used, allowing visual confirmation through conventional fluorescently labeled siRNA. After a 6 h incubation period, the cells were switched to fresh medium and cultured for an additional two days. The effectiveness of the gene silencing was evaluated through RT-PCR analysis. In cells treated with 80 pM of siRNA, there was a 90% reduction in BK_Ca_ mRNA levels three days post-transfection.

### 4.16. Electrophysiological Studies

Electrophysiological recordings were conducted with a patch clamp amplifier (Axopatch 200, Axon Instruments, Union City, CA, USA) at room temperature. Single-channel currents were digitized using a VR10 digital data recorder (Instrutech, Great Neck, NY, USA) and recorded onto videotape. The signals were filtered at 2 kHz with an 8-pole Bessel filter (−3 dB; Frequency Devices, Haverhill, MA, USA), and then transferred to a computer via a Digidata 1322A interface (Axon Instruments) at a sampling rate of 20 kHz. The channel opening detection threshold was set at 50%. Whole-cell currents were recorded post-capacitative transient cancellation. Analysis of both whole-cell and single-channel currents was conducted using the pCLAMP software (Version 8). For single-channel analysis, the filter’s dead time was set at 100 µs (0.3/cutoff frequency), meaning events shorter than 50 µs would not be detected. Channel activity (NPo, where N is the number of channels in the patch and Po is the open probability of a channel) was derived from approximately 1–2 min of current recording. The displayed single-channel current tracings in the figures were filtered at 2 kHz. In cell-attached and excised patch experiments, both pipette and bath solutions contained the following (in mM): 150 KCl, 1 MgCl_2_, 5 EGTA, and 10 HEPES, with a pH of 7.3. For whole-cell recordings, the bath solution comprised (in mM) 135 NaCl, 5 KCl, 1 CaCl_2_, 1 MgCl_2_, 5 glucose, and 10 HEPES, while the pipette solution was the same as that used for single-channel recording. The pH was adjusted to 7.3 using either HCl or KOH (NaOH).

### 4.17. Statistical Analysis

The data are represented as the mean ± SD. The Student’s *t*-test or Mann–Whitney test was used with a *p* < 0.05 as the criterion for significance.

## 5. Conclusions

Our findings showed that inhibition of BK_Ca_ channels enhanced migration and proliferation of NHEKs during wound healing, suggesting a novel therapeutic target for improving wound healing outcomes. The downregulation or inhibition of BK_Ca_ channels can be a potential strategy to accelerate cutaneous wound healing.

## 6. Patents

Korean patent No. 10-0855635 (invention name: composition for promoting wound healing).

## Data Availability

The data related to this study are not currently stored in a publicly accessible repository. However, the authors are willing to provide the data upon reasonable request. Requests for access to the data can be directed to Dawon Kang (dawon@gnu.ac.kr).
